# Supplementary material for: In vivo evolution of an emerging zoonotic bacterial pathogen in an immunocompromised human host
Source: Nat Commun. 2021 Jul 23;12:4495. doi: 10.1038/s41467-021-24668-7 (PMC8302680; doi:10.1038/s41467-021-24668-7)
Supplement: Supplementary file 1 — Supplementary Information [file 41467_2021_24668_MOESM1_ESM.pdf]

SUPPLEMENTARY INFORMATION

In Vivo Evolution of an Emerging Zoonotic Bacterial Pathogen in an Immunocompromised Human Host

Launay A[1], Wu CJ[1], Dulanto Chiang A[1], Youn JH[2], Khil PP[1,2], Dekker JP[1,2#]

[1]Bacterial Pathogenesis and Antimicrobial Resistance Unit, LCIM, NIAID, Bethesda, MD; [2]Dept. Laboratory Medicine, NIH Clinical Center, Bethesda, MD

#Address correspondence to John Dekker at john.dekker@nih.gov

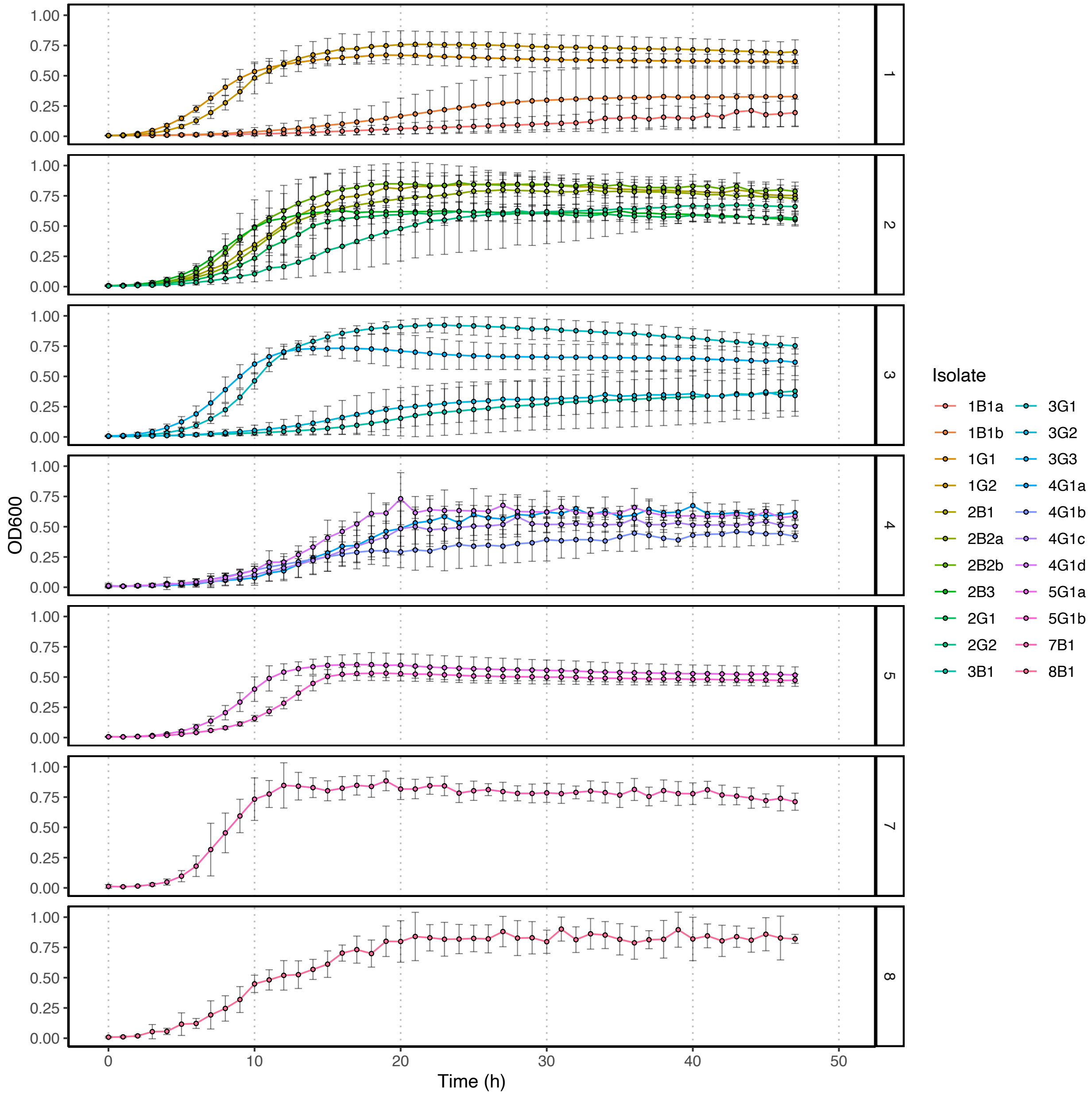

Supplementary Figure 1. Growth curves of patient *B. hinzii* isolates show significant variability. Growth in rich media at 37°C as measured by optical density at 600 nm. Points represent mean OD 600 value of replicates plotted on a linear scale, and the error bars indicate the standard deviation. Isolates are grouped by original date of isolation represented as culture number. Culture number 1: 1B1a (n = 7), 1B1b (n = 6), 1G1 (n = 3), 1G2 (n = 5); Culture number 2: 2B1 (n = 7), 2B2a (n = 7), 2B2b (n = 5), 2B3 (n = 6), 2G1 (n = 7), 2G2 (n = 6); Culture number 3: 3B1 (n = 7), 3G1 (n = 6), 3G2 (n = 7), 3G3 (n = 7); Culture number 4: 4G1a (n = 7), 4G1b (n = 7), 4G1c (n = 7), 4G1d (n = 7); Culture number 5: 5G1a (n = 5), 5G1b (N = 5); Culture number 7: 7B1 (N = 7); Culture number 8: 8B1 (N = 7). Isolate identities are given in legend at right. Note the extreme degree of variability in time to reach stationary phase, ranging from ~10 hr (7B1) to >25 hr (1B1a and 1B1b).

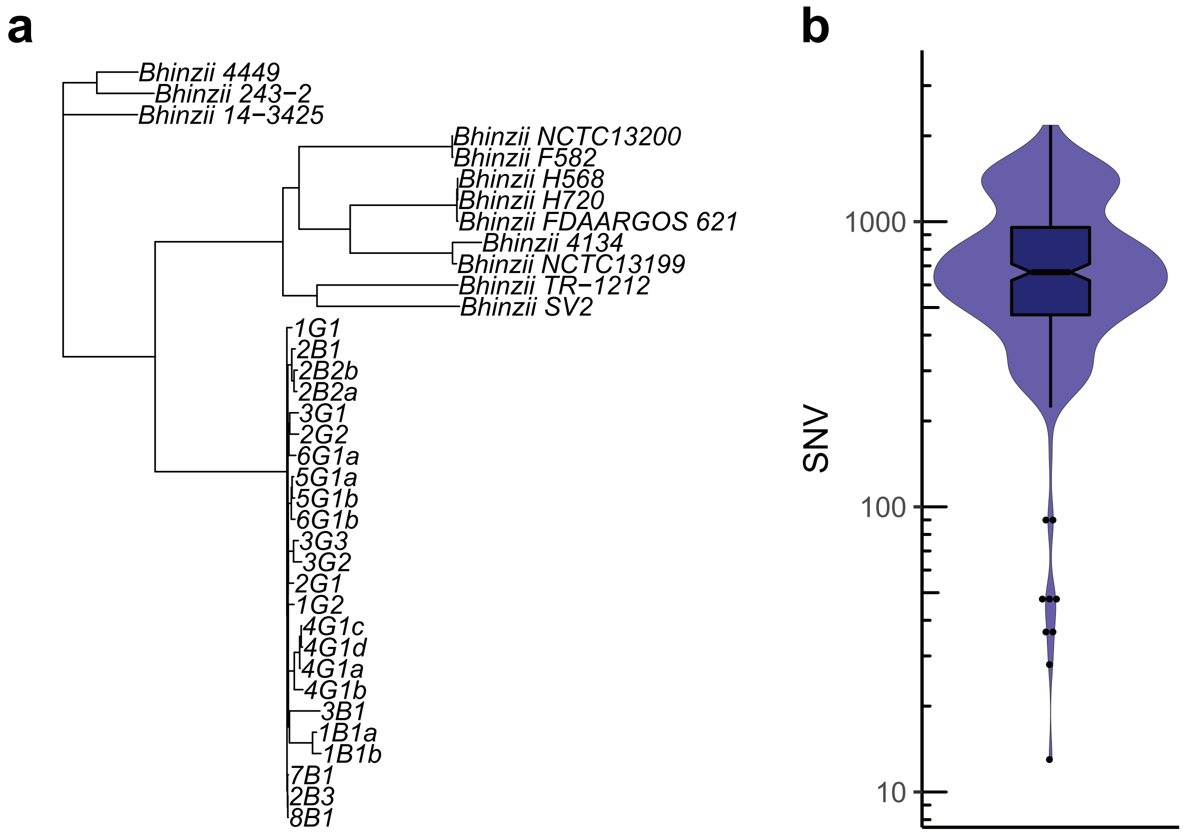

Supplementary Figure 2. ***B. hinzii*** **isolates cultured from patient form a single lineage with a striking range of divergence. a,** Core genome phylogeny generated with RAxML using the GTRGAMMA model, constructed from patient isolates in this study and publicly available *B. hinzii* genomes (downloaded from NCBI). The patient isolates are seen to form a distinct clade. **b**, Violin plot demonstrating pairwise divergence values for patient *B. hinzii* isolates (number of comparisons N = 276). Horizontal lines inside boxes indicate median values, the upper and lower edges correspond to the 25th and 75th percentiles, and whiskers extend to 1.5 × IQR. Outlier values outside of 1.5 × IQR represented as dots. A large range of pairwise divergence values is observed.

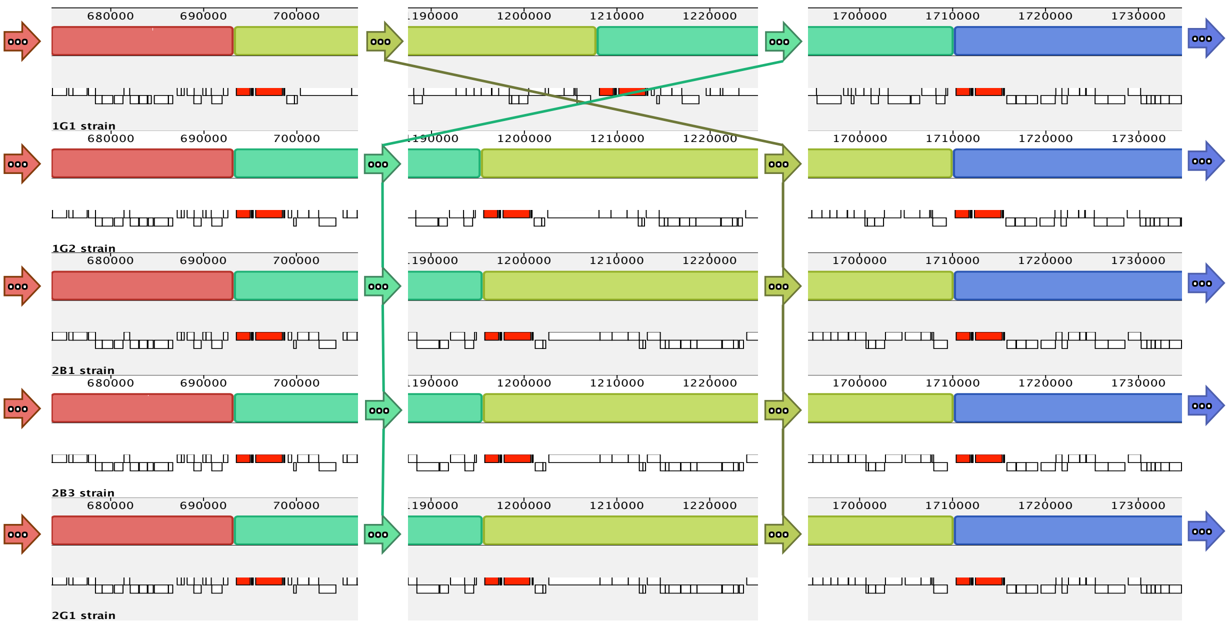

Supplementary Figure 3. The genome of isolate 1G1 contains a 500 Kb translocation and a 53 Kb deletion. Comparison (from top to bottom) of a region of genome from isolates 1G1, 1G2, 2B1, 2B3, and 2G1. A 500kb translocation is visible in 1G1. The translocated segment is flanked by rRNA sequences, highlighted in red in the annotation below each genome segment, suggesting a potential mechanism of recombination.

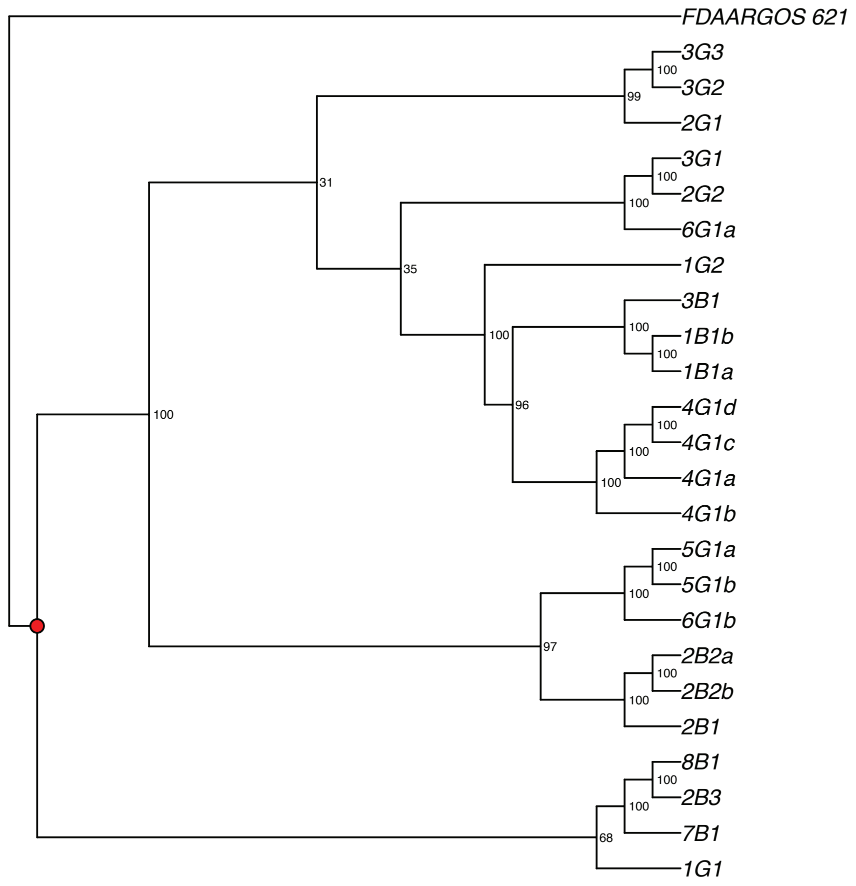

Supplementary Figure 4. Rooted cladogram of patient isolates. ML core genome phylogeny of patient isolates and FDA_Argos 621 as *B. hinzii* outgroup generated with RAxML using the GTRGAMMA model. The lowest common ancestor (LCA) of the patient clade is indicated with the red dot. The numbers at the node of the tree indicate bootstrap support.

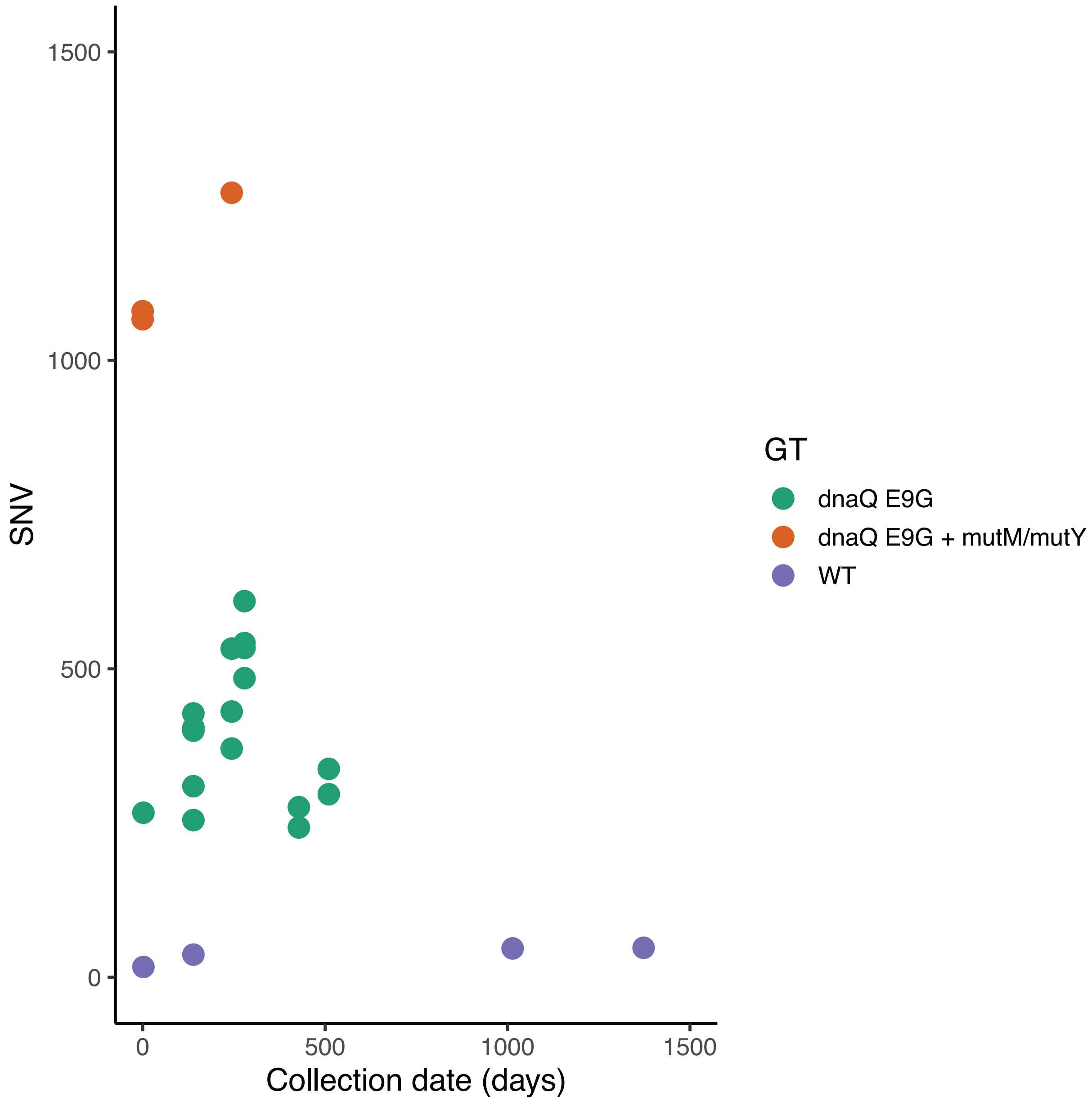

Supplementary Figure 5. Divergence of patient isolates from LCA versus collection date. The number of SNVs present in different patient isolates collected on different days is plotted. Genotype class is distinguished by color as indicated in the legend at right.

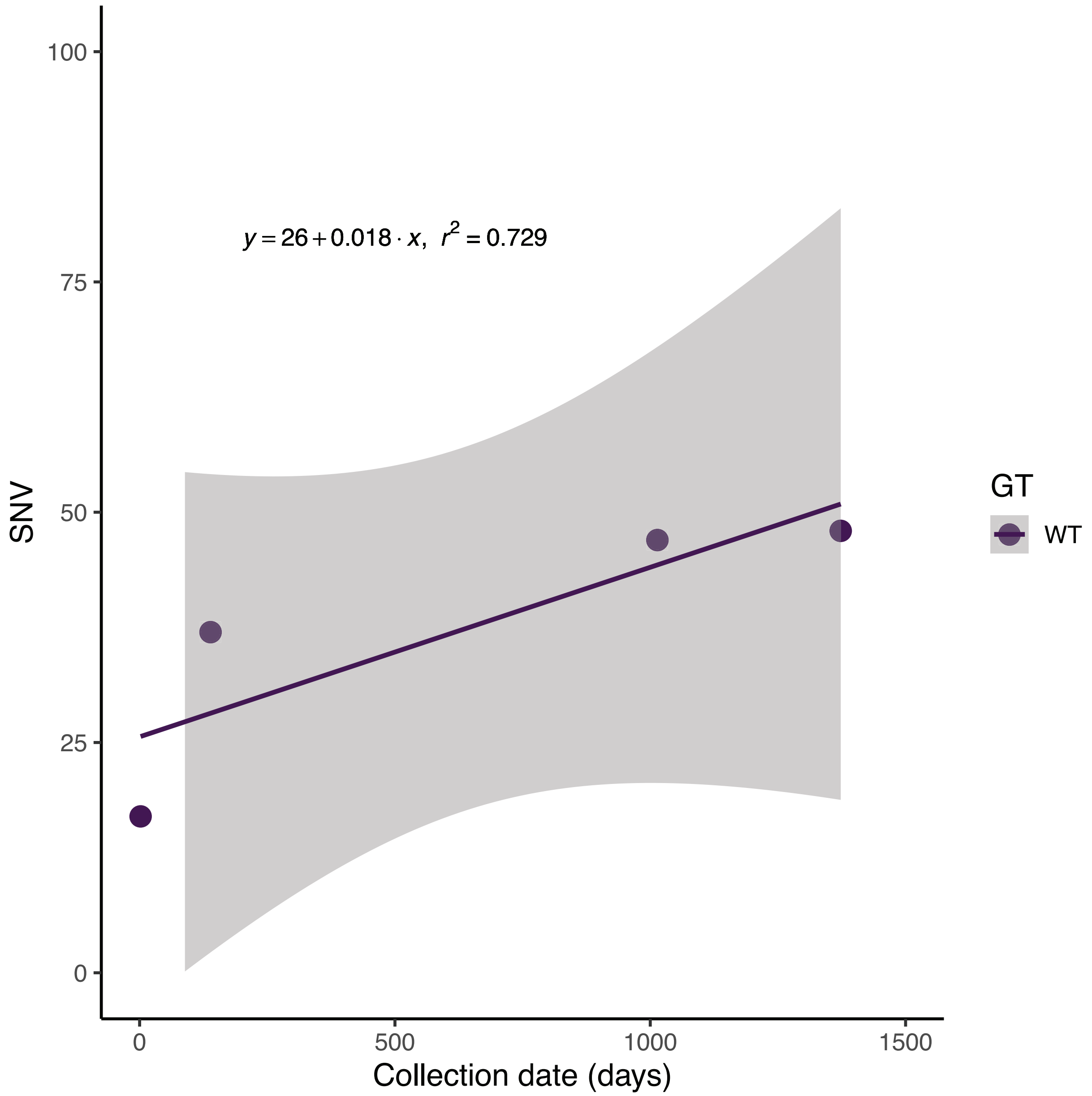

Supplementary Figure 6. Divergence of patient isolates with wild type DnaQ gene from LCA versus collection date. Also shown is a linear regression fit (+/- 95% CI).

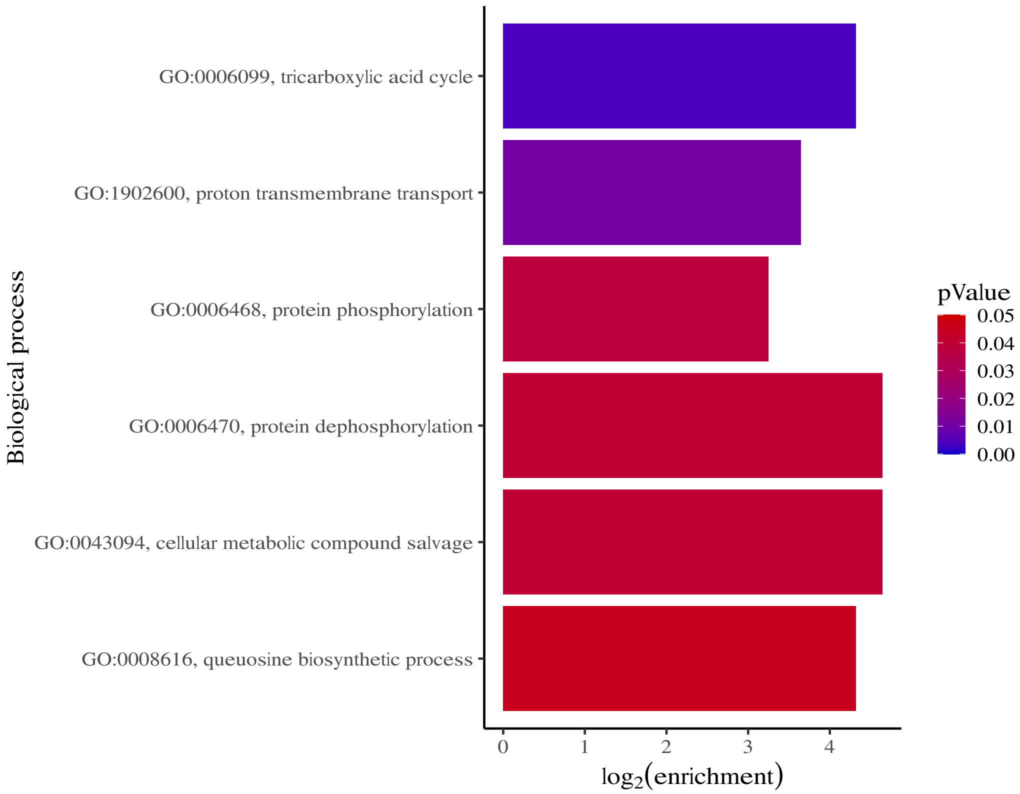

Supplementary Figure 7. Gene ontology classification of the 25 variant targets that are shared by all DnaQ E9G proof-reading deficient isolates. TopGO weighted GO analysis of the 25 variant targets shared by all DnaQ E9G isolates with $p<0.05$ (one-tailed Fisher's exact test) demonstrates functional enrichment of a number of cellular processes, with strongest enrichment seen in tricarboxylic acid cycle targets.

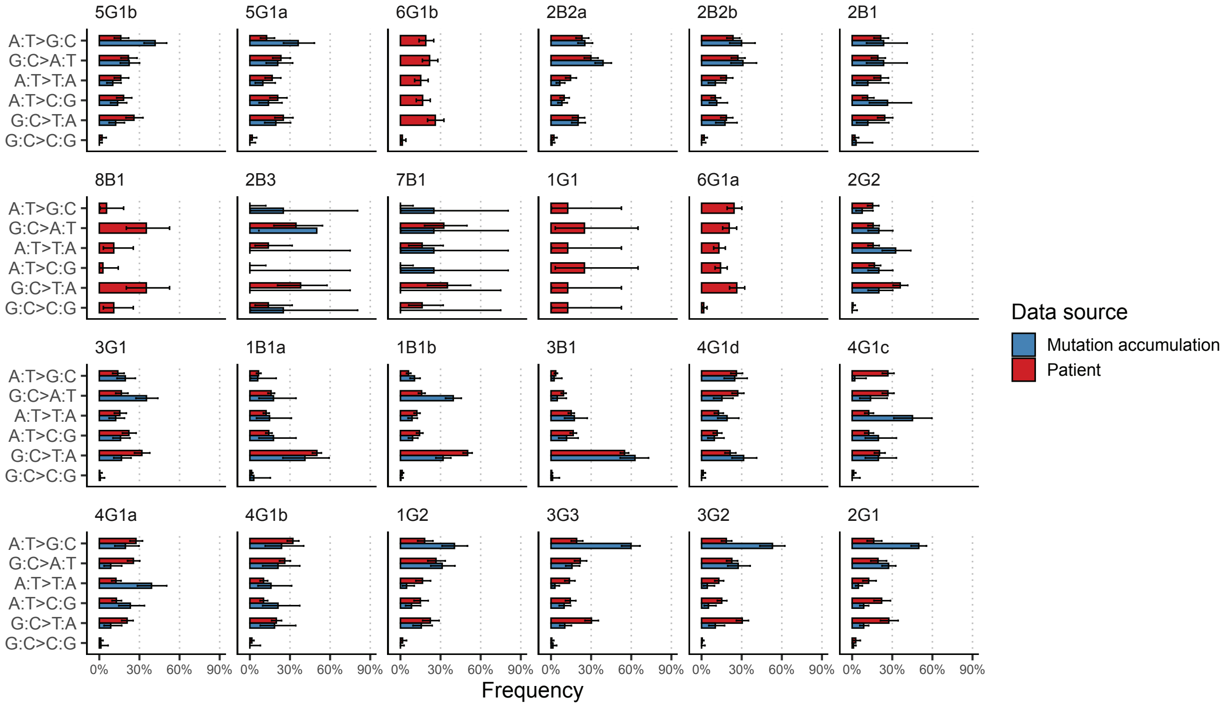

Supplementary Figure 8. Lineage-specific mutational spectra in patient isolates largely reflects the mutational spectra observed in *in vitro* mutation accumulation experiments. Comparison of mutational spectra present in patient isolates (red) with mutational spectra observed in *in vitro* mutation accumulation experiment, performed with the same isolates (blue). For the analysis, all data were aggregated over all set of technical replicates (see Methods for details). Bars represent the binomial estimates of frequency of each substitution type in each isolate, and the lines represent Clopper and Pearson 95th percentile confidence interval estimates.

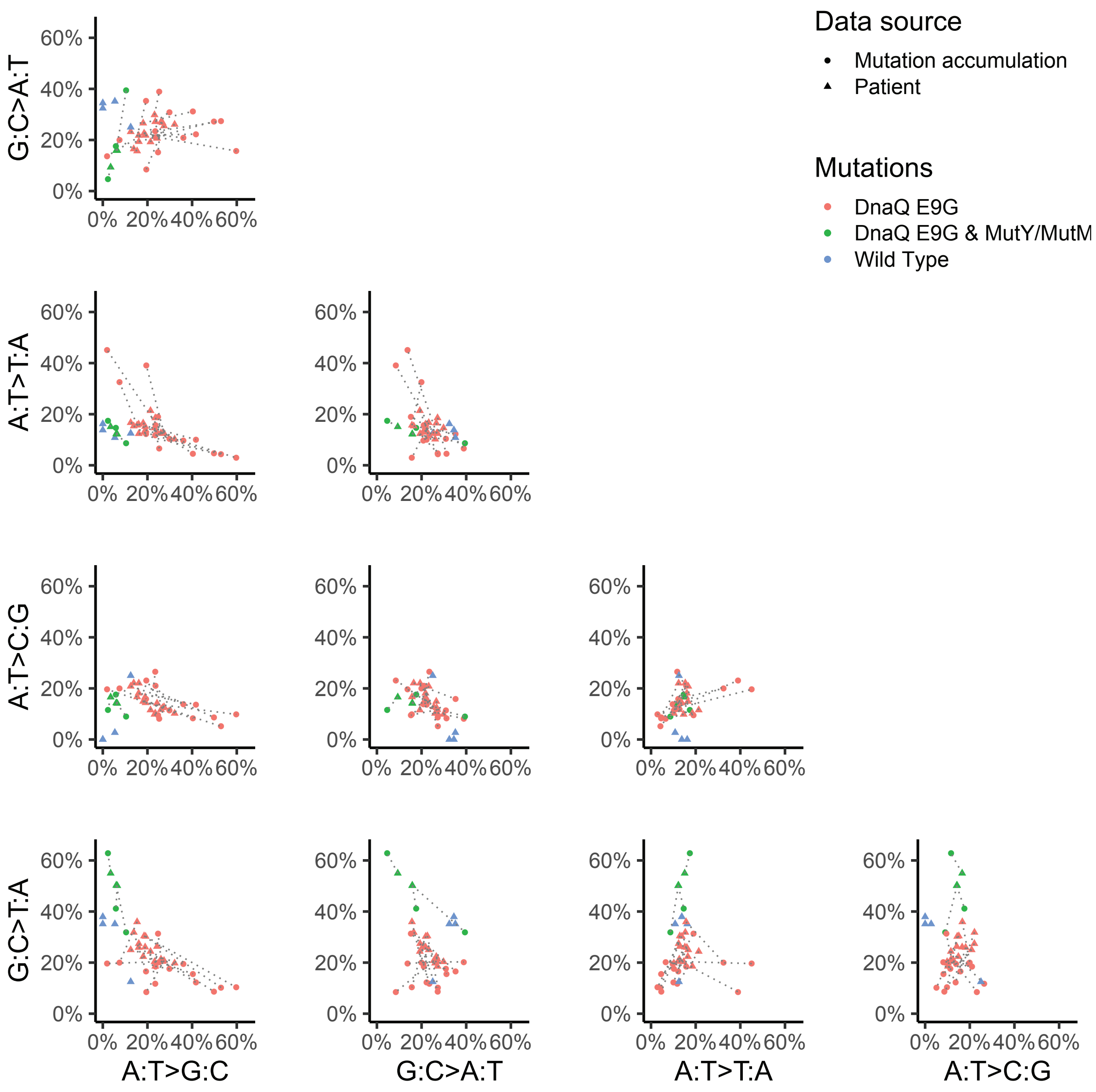

Supplementary Figure 9. Comparison of mutational spectra in patient isolates and mutational spectra in *in vitro* mutation accumulation experiments. Mutational spectra in patient isolates are shown as triangles, and mutational spectra observed in *in vitro* mutation accumulation experiments performed on same isolates are shown as circles, connected by a dashed line.

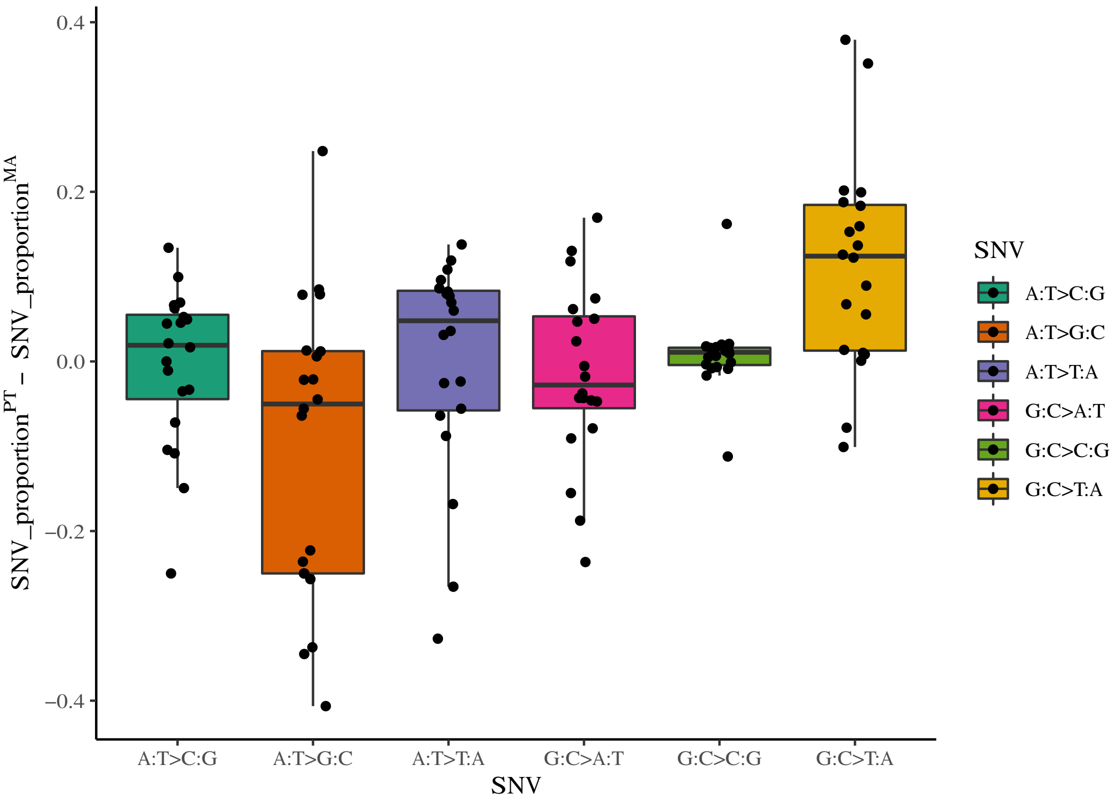

Supplementary Figure 10. Comparison of substitution categories in patient isolates and *in vitro* mutation accumulation experiments. Box plot shows, for each category of mutations, the difference between the substitution frequency seen in patient isolates and the frequency seen in the mutation accumulation experiment (n= 6 × 20). Horizontal lines inside boxes indicate median values, the upper and lower edges correspond to the 25th and 75th percentiles, and whiskers extend to 1.5 × IQR. The frequency of C:G>T:A substitutions is notably elevated in patient isolates, consistent with an oxidative mutagenesis signature.

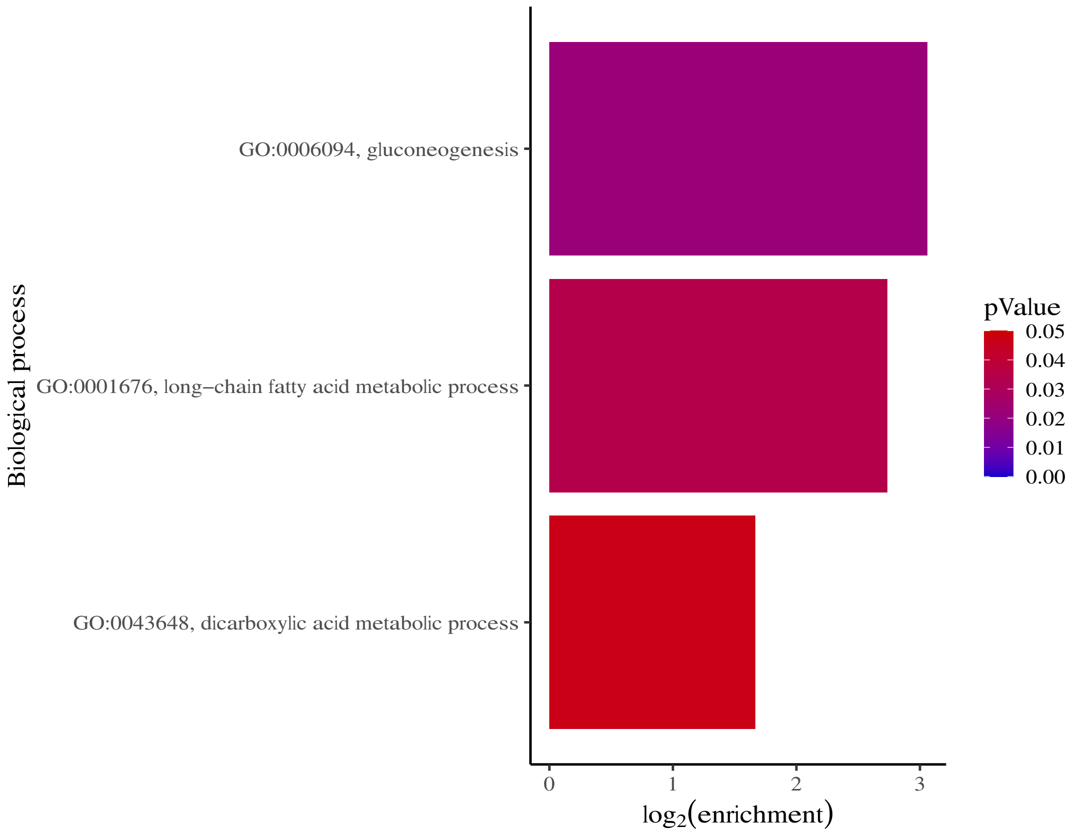

Supplementary Figure 11. Gene ontology classification of mutational targets identified in patient isolates. TopGO weighted analysis of targets demonstrating repeated independent mutations with $p<0.05$ (one-tailed Fisher's exact test) demonstrates enrichment of targets in gluconeogenesis, long-chain fatty acid metabolic processes and dicarboxylic acid metabolic processes.

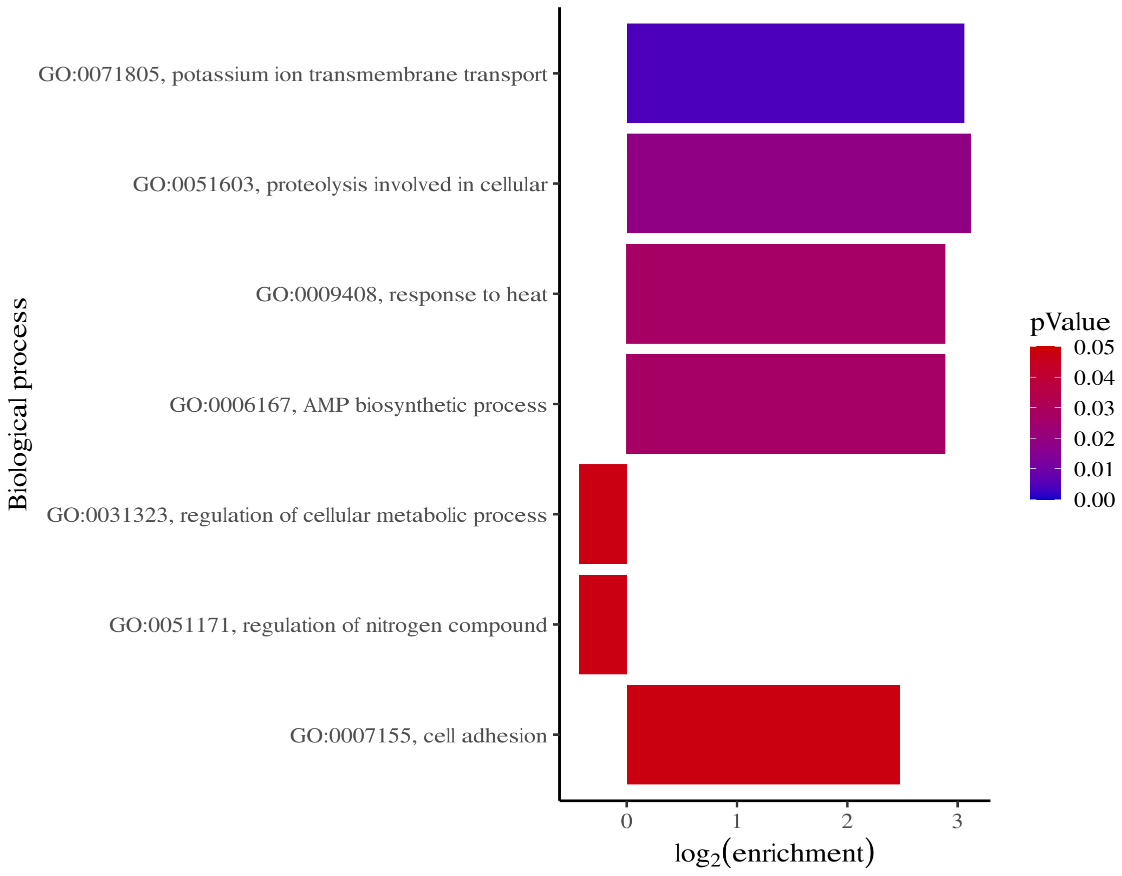

Supplementary Figure 12. Gene ontology classification of pseudogenes identified in patient isolates. TopGO weighted analysis of identified pseudogenes with $p<0.05$ (one-tailed Fisher's exact test) demonstrates functional enrichment of targets in proteolysis, response to heat, and cell adhesion.

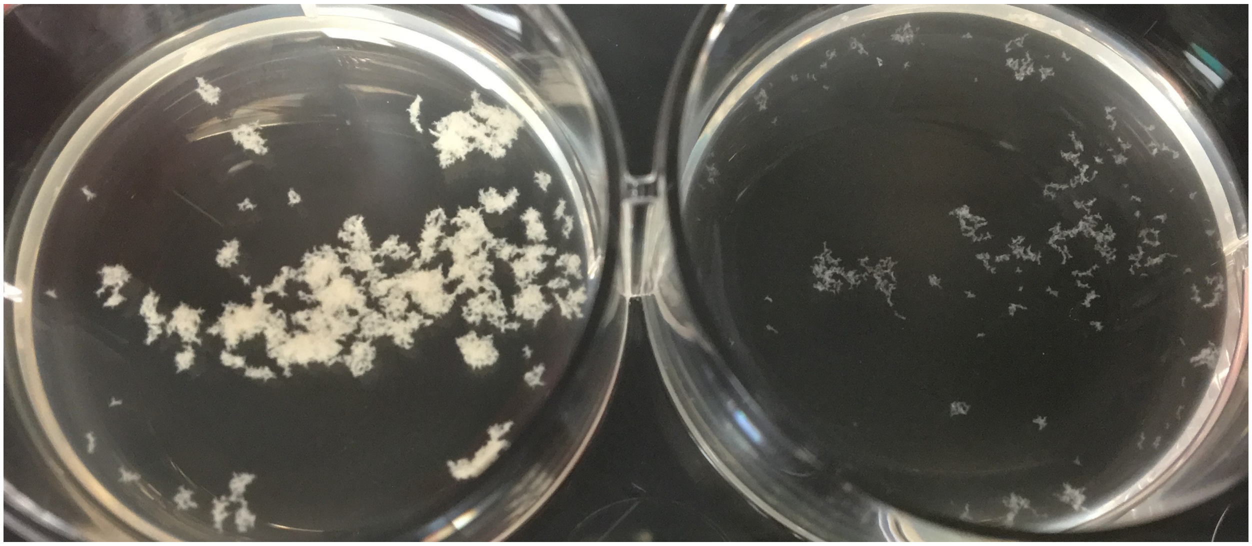

Supplementary Figure 13. Macroscopic aggregation in liquid culture. Cultures from the 8B1 (left) and 2B3 (right) lineages demonstrate prominent growth as macroscropic aggregates when grown in broth culture.

Supplementary Table 1. Colony morphologies observed for patient *B. hinzii* isolates

Phenotype/Isolate	1B1a	1B1b	1G1	1G2	2B1	2B2a	2B2b	2B3	2G1	2G2	3B1	3G1	3G2	3G3	4G1a	4G1b	4G1c	4G1d	5G1a	5G1b	8B1
Mucoid (M) / Colonies (C)	M/C	M/C	M/C	C	M	C	M	C	C	C	M/C	C	M/C	C	C	M	C	C	C	C	C
Colony Size	+	++	++	+/++	NA	+/++	NA	+/++	+/++	++	+	++	+	++	++	NA	++	++	++	+/++	+
Opaque (O) / Translucent (T)	T	O/T	O	O	O/T	O	O	O	O	O	O/T	O	O/T	O	O	O	O	O	O	O	O

NA = Not Applicable. M/C = mixture of mucoid and single colonies. +/++ = mixture of two colony sizes. O/T = mixture of opaque and translucent.

Supplementary Table 2. Antimicrobial Susceptibility profiles for patient *B. hinzii* isolates

Drug/Isolate	1G1	1G2	2G1	2B1	2B3	3G2	3G3
Doxycycline	2	0.5	8	8	4	-	-
Minocycline	-	-	8	16	2	-	-
Tetracycline	-	-	8	32	4	-	-
Tigecycline	-	-	2	2	2	0.25	0.5
Pip/Tazo	≤4/4	≤4/4	>128/4	>128/4	>128/4	-	-
Azithromycin			-	-	-	1	8
Polymixin B	-	-	1	≤0.25	2	-	-
Levofloxacin	>8	8	>16	16	>16	-	-
Trimeth/Sulfa	>8/152	>8/152	>8/152	27851	>8/152	-	-
Ceftazidime	-	-	>128	>128	64	-	-
Ertapenem	-	-	>8	4	4	-	-
Imipenem	-	-	4	2	2	1	1
Meropenem	8	8	>8	4	4	4	8
Amikacin	4	4	32	16	8	-	-
Gentamycin	2	2	8	8	4	-	-
Tobramycin	8	8	>16	>16	16	-	-
Cefepime	-	-	32	>32	>32	-	-
Colistin	-	-	1	≤0.25	2	<=0.25	4

Pip/Tazo = Piperacillin/Tazobactam; Trimeth/Sulfa = Trimethoprim/Sulfamethoxazole

Supplementary Table 3. Sequencing and assembly information for patient *B. hinzii* isolates

Isolates	Sequencing technology	Contigs	Total length	GC (%)	Cov	N50
1G1	PacBio	1	4845358	66.98	108.28	4845358
1G2	PacBio	1	4898905	67.01	161.19	4898905
2B1	PacBio	1	4899058	67.01	161.13	4899058
2B3	PacBio	1	4899031	67.01	227.9	4899031
2G1	PacBio	1	4898869	67.01	137.45	4898869
1B1a	Illumina	51	4874957	67.03	81.69	200971
1B1b	Illumina	52	4875262	67.03	87.28	228726
1G1	Illumina	30	4819330	67.02	70.4	422032
1G2	Illumina	32	4873616	67.04	75.99	424690
2B1	Illumina	27	4874913	67.05	114.5	424690
2B2a	Illumina	34	4872885	67.05	244.17	424267
2B2b	Illumina	35	4872434	67.05	138.38	334930
2B3	Illumina	28	4874194	67.05	146.11	424690
2G1	Illumina	31	4874440	67.05	138.85	424690
2G2	Illumina	30	4874045	67.04	197.13	424264
3B1	Illumina	32	4870893	67.03	194.82	424691
3G1	Illumina	70	4867756	67.04	63.44	119745
3G2	Illumina	31	4874477	67.05	109.9	424264
3G3	Illumina	29	4873819	67.05	173.99	424264
4G1a	Illumina	36	4873355	67.05	40.85	334930
4G1b	Illumina	30	4874646	67.05	137.18	434284
4G1c	Illumina	33	4874276	67.05	185.81	434273
4G1d	Illumina	36	4874953	67.04	81.77	370497
5G1a	Illumina	31	4874384	67.05	53.52	425144
5G1b	Illumina	30	4873668	67.04	61.56	424691
6G1a	Illumina	29	4874601	67.05	75.78	424690
6G1b	Illumina	32	4874287	67.05	101.96	424690
7B1	Illumina	73	4867858	67.04	42.53	150297
8B1	Illumina	64	4829741	67.1	50.66	139051
ATCC_51783	Illumina	69	4997723	65.97	67.09	118470
ATCC_51784	Illumina	81	4867486	67.51	66.99	116274

Supplementary Table 4. Deletions in the genomes of patient isolates

Position	Length (bp)	Gene name	Locus tag	Product	In isolates
522746	267	adoK	A_00509	Adenosine kinase	5G1a,5G1b,6G1b
1044661	117	NA	A_00961	hypothetical protein	1G2
1479787	180	ltaE to A_01369	A_01368 to A_01369	2 genes	4G1a,4G1b,4G1c,4G1d
2144786	53478	gabR_2 to hsrA_2	A_02003 to A_02051	49 genes	1G1
3072620	1530	NA	A_02898	hypothetical protein	3B1
3152552	455	A_02961	A_02961	hypothetical protein	3B1
3823276	38482	NA	A_03624 to A_03675	52 genes	8B1,8B2
4566359	428	trkH	A_04354	Trk system potassium uptake protein TrkH	1B1a,1B1b
4573054	333	rsmB_2	A_04359	Ribosomal RNA small subunit methyltransferase B	5G1a,5G1b,6G1b
4722262	285	steT	A_04511	Serine/threonine exchanger SteT	3B1

Supplementary Table 5. Mutational spectra from patient isolates and mutation accumulation experiments.

Patient Isolate SNVs	5G1b	5G1a	6G1b	2B2a	2B2b	2B1	8B1	2B3	7B1	1G1	6G1a	2G2	3G1	1B1a	1B1b	3B1	4G1d	4G1c	4G1a	4G1b	1G2	3G3	3G2	2G1
A:T>G:C	31	21	40	73	71	50	2	0	0	1	60	47	37	56	51	35	105	110	99	148	34	64	76	30
G:C>A:T	42	39	46	94	82	45	13	10	12	2	51	48	44	137	134	93	109	110	92	120	50	73	93	36
A:T>T:A	31	28	32	46	56	50	4	4	6	1	32	48	41	105	104	150	51	51	45	47	31	46	53	23
A:T>C:G	35	35	35	31	31	27	1	0	0	2	35	51	59	122	121	165	47	51	46	47	28	48	62	41
G:C>T:A	50	42	55	64	56	57	13	11	13	1	65	110	85	433	425	547	86	84	75	91	42	101	125	51
G:C>C:G	4	3	3	7	6	5	4	4	6	1	4	2	1	11	11	5	5	5	4	7	3	2	2	5
**Mutational Accumulation**	**5G1b**	**5G1a**	**6G1b**	**2B2a**	**2B2b**	**2B1**	**8B1**	**2B3**	**7B1**	**1G1**	**6G1a**	**2G2**	**3G1**	**1B1a**	**1B1b**	**3B1**	**4G1d**	**4G1c**	**4G1a**	**4G1b**	**1G2**	**3G3**	**3G2**	**2G1**
A:T>G:C	58	26		65	29	8		1	1			6	27	2	29	2	26	1	16	9	44	122	62	150
G:C>A:T	31	15		100	30	8		2	1			16	49	6	109	4	16	7	7	8	34	32	32	82
A:T>T:A	14	7		17	10	4		0	1			26	17	5	24	15	20	23	32	6	5	6	5	14
A:T>C:G	19	10		21	11	9		0	1			16	22	6	25	10	10	10	19	8	9	20	6	26
G:C>T:A	17	14		52	17	4		0	0			16	23	14	88	54	33	10	7	7	17	21	12	26
G:C>C:G	0	0		2	0	1		1	0			0	1	1	1	1	0	0	1	0	0	3	0	3

Numbers of variants in for each category of substitutions are given.

Supplementary Table 6. Calculation of number of passages that would be required to produce the observed number of mutations in each isolate based on *in vitro* mutation rates

Isolate	SNVs	SNV per_division	Divisions	95th CI divisions	Passages
1B1a	142	0.3125	454.4	325.4-655.9	16.7
1B1b	132	2.5368	52	46.3-58.7	1.9
2B2a	126	1.3498	93.3	82.7-105.8	3.4
2B2b	117	0.5944	196.8	161.5-242.6	7.2
4G1a	19	0.0773	245.8	198-309	9
4G1b	317	0.0437	7261	5290.5-10259.9	266.9
4G1c	18	0.0647	278.4	211.8-373.9	10.2
4G1d	10	0.2145	46.6	38.5-57	1.7
5G1a	58	0.3782	153.4	121.9-195.9	5.6
5G1b	90	0.73	123.3	104.5-146.6	4.5

Supplementary Table 7. Additional genomes used in this study

Strain	Accession	Genome size	Host	Country	Collection
F582	CP012076	4912977	H. sapiens	USA: WA	1994
FDAARGOS_621	CP044059	4860452	H. sapiens	USA: MI	NA
H568	CP012077	4859884	H. sapiens	USA: AR	2010
H720	CP024172	4859886	NA	USA: MN	2010
NCTC13199	LR134382	5033537	Chicken	Australia	1900/1993
14-3425	CP021398	4885078	Turkey	USA: NC	2019
243-2	CP021400	4811419	Turkey	USA: MN	1997
NCTC13200	LT906461	4913806	NA	NA	1900/2007
SV2	CP021395	4809490	Turkey	USA: VA	2006
4134	CP021396	5042960	Turkey	USA: MN	NA
4449	CP021397	4898820	Turkey	USA: MN	NA
TR-1212	CP021399	4930681	Turkey	USA	NA
