# Supplementary material for: In vivo evolution of an emerging zoonotic bacterial pathogen in an immunocompromised human host
Source: Nat Commun. 2021 Jul 23;12:4495. doi: 10.1038/s41467-021-24668-7 (PMC8302680; doi:10.1038/s41467-021-24668-7)
Supplement: Supplementary file 3 — Description of Additional Supplementary Files [file 41467_2021_24668_MOESM3_ESM.pdf]

Description of Additional Supplementary Files

File Name: Supplementary Data 1
Description: Mutations present in patient isolates relative to LCA

File Name: Supplementary Data 2
Description: Statistical analysis of targets with multiple independent mutations

File Name: Supplementary Data 3
Description: dN/dS calculations

File Name: Supplementary Data 4
Description: Pseudogenes identified in patient isolates
