# Supplementary material for: In vivo evolution of an emerging zoonotic bacterial pathogen in an immunocompromised human host
Source: Nat Commun. 2021 Jul 23;12:4495. doi: 10.1038/s41467-021-24668-7 (PMC8302680; doi:10.1038/s41467-021-24668-7)
Supplement: Supplementary file 8 — Reporting Summary [file 41467_2021_24668_MOESM8_ESM.pdf]

nature research

Corresponding author(s): John Dekker
Last updated by author(s): May 31, 2021

Reporting Summary

Nature Research wishes to improve the reproducibility of the work that we publish. This form provides structure for consistency and transparency in reporting. For further information on Nature Research policies, see our Editorial Policies and the Editorial Policy Checklist.

Statistics

For all statistical analyses, confirm that the following items are present in the figure legend, table legend, main text, or Methods section.

n/a	Confirmed	
☐	☒	The exact sample size (*n*) for each experimental group/condition, given as a discrete number and unit of measurement
☐	☒	A statement on whether measurements were taken from distinct samples or whether the same sample was measured repeatedly
☐	☒	The statistical test(s) used AND whether they are one- or two-sided<br>*Only common tests should be described solely by name; describe more complex techniques in the Methods section.*
☒	☐	A description of all covariates tested
☐	☒	A description of any assumptions or corrections, such as tests of normality and adjustment for multiple comparisons
☐	☒	A full description of the statistical parameters including central tendency (e.g. means) or other basic estimates (e.g. regression coefficient) AND variation (e.g. standard deviation) or associated estimates of uncertainty (e.g. confidence intervals)
☐	☒	For null hypothesis testing, the test statistic (e.g. *F*, *t*, *r*) with confidence intervals, effect sizes, degrees of freedom and *P* value noted<br>*Give P values as exact values whenever suitable.*
☒	☐	For Bayesian analysis, information on the choice of priors and Markov chain Monte Carlo settings
☒	☐	For hierarchical and complex designs, identification of the appropriate level for tests and full reporting of outcomes
☒	☐	Estimates of effect sizes (e.g. Cohen's *d*, Pearson's *r*), indicating how they were calculated

Our web collection on statistics for biologists contains articles on many of the points above.

Software and code

Policy information about availability of computer code

Data collection	Software was used for data analysis as below, but not for data collection.
Data analysis	All custom scripts used for calculations are posted with zenodo https://doi.org/10.5281/zenodo.4894884.<br>Software used: Prokka v1.14.6, PHAST v1.4, SAMtools v1.9, FreeBayes v1.3.1, BLAST v2.10.0, topGO v2.38.1, BEDtools v2.72.1, SPAdes v3.14.0, Roary v3.13.0, Snippy v4.4.0, R v4.0.0, ape v5.4, blast2go v5.2.5, QUAST v5.0.2, SnpEff v5.0, RAxML v8.2.12, BWA 0.7.17, samclip v0.2, Vt Normalize v0.5, VCFtools v0.1.16, Breseq v0.35.1,InterProScan v5.42-78.0 .

For manuscripts utilizing custom algorithms or software that are central to the research but not yet described in published literature, software must be made available to editors and reviewers. We strongly encourage code deposition in a community repository (e.g. GitHub). See the Nature Research guidelines for submitting code & software for further information.

Data

Policy information about availability of data

All manuscripts must include a data availability statement. This statement should provide the following information, where applicable:

- Accession codes, unique identifiers, or web links for publicly available datasets
- A list of figures that have associated raw data
- A description of any restrictions on data availability

Sequencing data have been deposited with NCBI under the accession number PRJNA625574. Source data are provided with this paper. Source data have also been deposited with Zenodo https://doi.org/10.5281/zenodo.4929939. Requests for bacterial isolates used in this study are subject to a negotiated Material Transfer Agreement with the NIH and US Government.

Field-specific reporting

Please select the one below that is the best fit for your research. If you are not sure, read the appropriate sections before making your selection.

[x] Life sciences [ ] Behavioural & social sciences [ ] Ecological, evolutionary & environmental sciences

For a reference copy of the document with all sections, see nature.com/documents/nr-reporting-summary-flat.pdf

Life sciences study design

All studies must disclose on these points even when the disclosure is negative.

Sample size	24 bacterial isolates collected from a single patient. The sample size was determined by the available set of bacterial isolates from the patient. All available isolates were used in the study to maximize information. No sample size calculations were performed, as all available bacterial isolates were used.
Data exclusions	No data were excluded. All data processing and filtering described in Methods.
Replication	Given that this is a descriptive study of within-host evolution of a single bacterial population in a single patient, verification of reproducibility of the main results is not possible for most parts of the study. Verification of reproducibility was relevant and possible was in the simulations performed to determine whether any genes demonstrated enrichment for mutations relative to null expectations. These simulations were repeated 100,000 times and quantified with detailed statistical tests as explained in the methods section of the manuscript.
Randomization	Randomization was not possible as this is a study of sequential evolution of all bacterial isolates collected from a single patient.
Blinding	Blinding was not relevant to this study as there were no results that would be relevant to blind the researchers to. The work involved sequencing of all genomes from the patient and studying the variants that occurred with evolution of the bacterial population in the patient.

Reporting for specific materials, systems and methods

We require information from authors about some types of materials, experimental systems and methods used in many studies. Here, indicate whether each material, system or method listed is relevant to your study. If you are not sure if a list item applies to your research, read the appropriate section before selecting a response.

Materials & experimental systems

n/a	Involved in the study	
x		Antibodies
x		Eukaryotic cell lines
x		Palaeontology and archaeology
x		Animals and other organisms
	x	Human research participants
x		Clinical data
x		Dual use research of concern

Methods

n/a	Involved in the study	
x		ChIP-seq
x		Flow cytometry
x		MRI-based neuroimaging

Human research participants

Policy information about studies involving human research participants

Population characteristics	Isolates collected during routine care of a single patient were studied. The patient was selected on the basis of two diagnoses: IL-12-beta 1 receptor deficiency and chronic infection with Bordetella hinzii. All other information from this single patient is de-identified (gender, age, etc) and not included in the manuscript and did not influence the study of the bacterial isolates.
Recruitment	The single patient from whom the bacterial isolates were collected was enrolled in NIH IRB protocol 93-I-0119 separately for the study and treatment of patients with certain immunodeficiencies. The patient received care under this protocol at the NIH Clinical Center that was separate from this study. Diagnostic clinical cultures were performed as part of routine standard-of-care management under this consented protocol, and only de-identified sub-cultured bacterial isolates were used in the work presented in this manuscript. The results of the work in this manuscript were not used for patient care and did not form the basis of a clinical intervention.
Ethics oversight	Informed written consent was obtained from the patient under NIH IRB protocol 93-I-0119 upon admission to the NIH Clinical Center, approved by NIH IRB committee. Diagnostic clinical cultures were performed as part of routine standard-of-care management under this consented protocol, and only de-identified sub-cultured bacterial isolates were used in the work presented in this manuscript. The results of the work in this manuscript were not used for patient care and did not form the basis of a clinical intervention. The work presented in this manuscript was thereby excluded from further NIH IRB review, on the basis of the fact that it was a study of a single case, involving only sequencing and analysis of bacterial isolates.

Note that full information on the approval of the study protocol must also be provided in the manuscript.
